# A Solitary Enchondroma of the Great Toe in an Adolescent Male: A Case Report

**DOI:** 10.7759/cureus.21772

**Published:** 2022-01-31

**Authors:** Shivam Patel, Siddharth Yadav, Sagar Gurnani, Parth Yadav, Bibin Selvin

**Affiliations:** 1 Orthopedics, Dr. D. Y. Patil Medical College, Hospital & Research Centre, Pune, IND

**Keywords:** tumor, chondrosarcoma, fibular strut graft, chondroma, enchondroma

## Abstract

Solitary enchondromas are benign and usually asymptomatic. Enchondromas are a form of cartilage tumor and have a higher chance of converting into chondrosarcoma. It is difficult to obtain a valid risk estimate, as this requires histopathology and MRI reports. A 17-year-old male presented with swelling over the left great toe since six months, which was insidious in onset, gradually progressive, and associated with intermittent dull aching type of pain. Physical examination revealed bony hard swelling of size 3 x 2.5cm over the left great toe. X-ray was suggestive of lytic lesions, scalloping of the cortex, and whorl of calcification. After confirming the diagnosis through MRI and histopathological examination, the decision was taken to remove whole of the proximal phalanx along with the tumor. Gap was filled up with fibular strut graft. Solitary enchondromas that are aggressively increasing in size should be treated surgically. Bone gap (between the first metatarsal and distal phalanx) caused after removing the tumor can be filled with bone graft or cement depending on the condition of the cortex.

## Introduction

Solitary enchondromas are usually benign but commonly asymptomatic. Enchondromas are a form of cartilage tumor that account for 3%-17% of all bone cancers [1,2] and around 20% of all cartilage tumors [1]. Enchondroma has the potential to convert into chondrosarcoma (CS), with a malignant transition rate ranging from 0% to 4.2% [3,4].To diagnose enchondromas, a histopathological investigation is required [1].

Enchondromas that have progressed to malignancy are classified as a low-grade/grade 1 CS by the World Health Organization. Because low-grade CS/ACT seldom metastasizes despite its local aggressiveness, the term "atypical cartilage tumor" (ACT) was coined [5,6]. The pitfall is the unintentional discovery of enchondroma on MRI when evaluating joint disease, which has been seen most frequently in the knee and shoulder [7-10]. However, an interobserver error by experienced pathologists and radiologists remains unknown [11].

## Case presentation

A 17-year-old male presented to the outpatient department with swelling in his left great toe since six months. The swelling was associated with a dull aching intermittent pain, without aggravating or relieving factors. It was also insidious during the onset, which gradually progressed.

**Figure 1:**
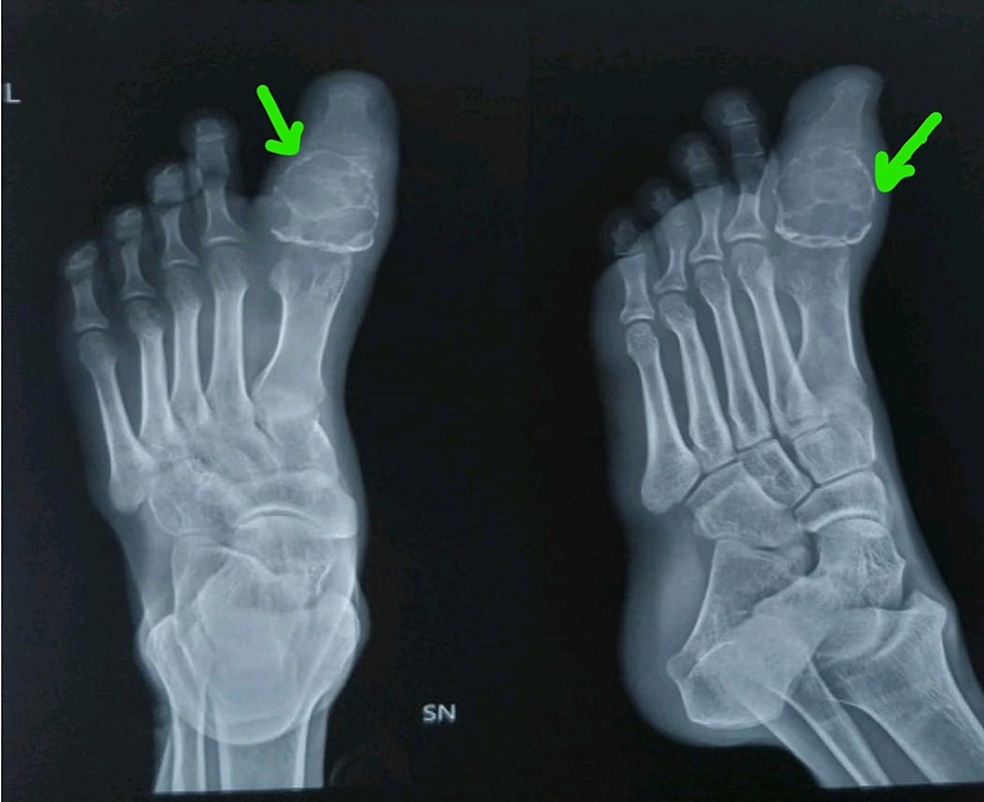
X-ray showing lytic lesions, scalloping of the cortex, and whorls of calcification.

**Figure 2:**
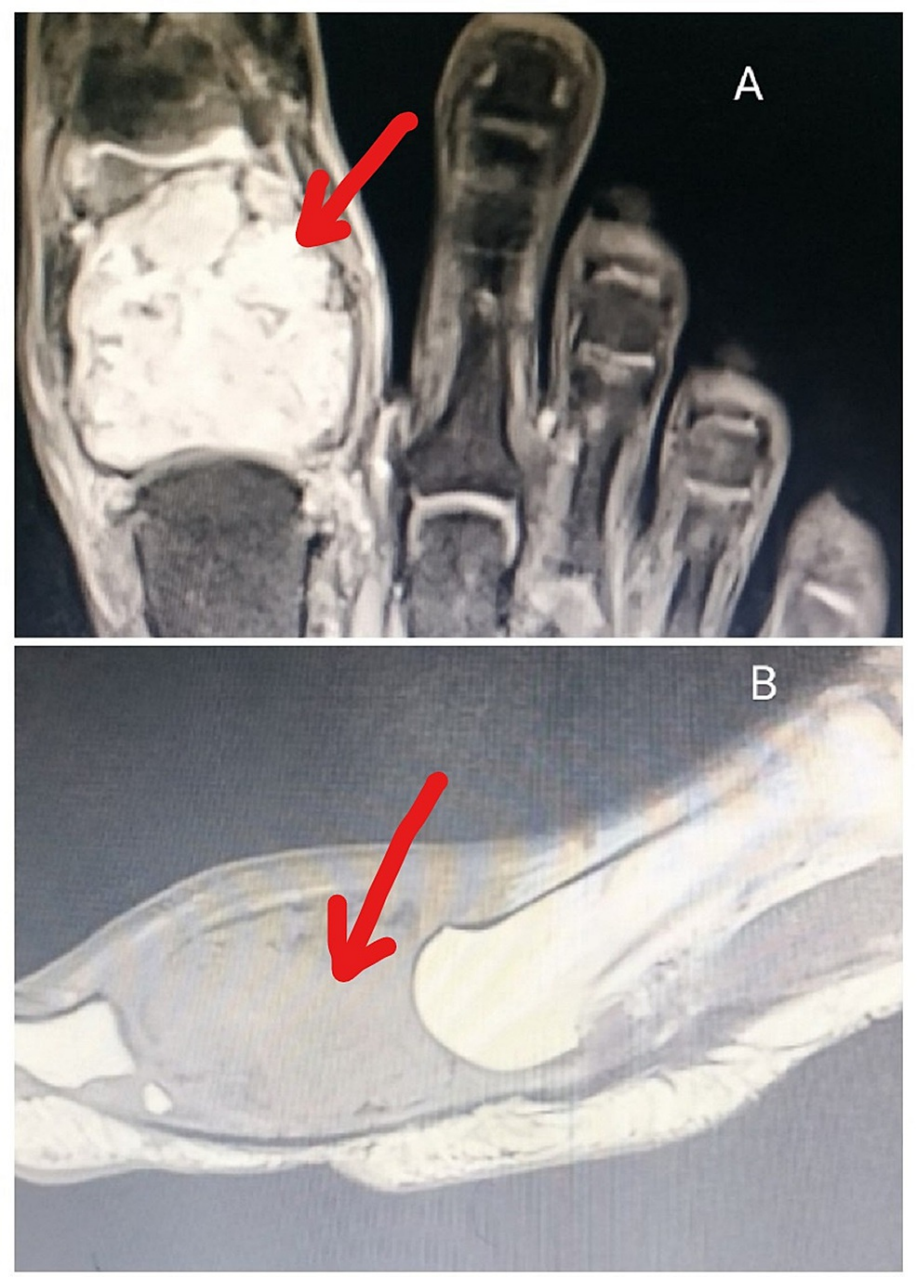
(A) MRI suggestive of a mass is hyperintense on FS-PD, with lesions involving the proximal and distal ends of the phalanx stippled calcification. (B) MRI suggestive of the expansion of the proximal phalanx of the great toe, hypointense on T1, with soft tissue edema and swelling around the proximal phalanx. FS-PD, fat-suppressed proton density; MRI, magnetic resonance imaging

Mild restriction at the motion of the left great toe (at the metatarsophalangeal [MTP] joint and interphalangeal joint) and a regular swelling over the dorsal aspect of the left great toe, which was hard in consistency without any neurovascular impairment, were observed. No presence of scars, sinuses, pigmentation, or any ulceration was observed over the swelling. Palpation of the swelling confirmed the presence of a bony hard swelling, nonpedunculated, and smooth surface of size 3 × 3 × 2.5 cm with ill-defined margins. The swelling was expansile and fixed to the skin. X-ray (Figure 1) and MRI (Figures 2A, 2B) were performed for further evaluation. The core needle biopsy was performed, which was suggestive of chondroma.

**Figure 3:**
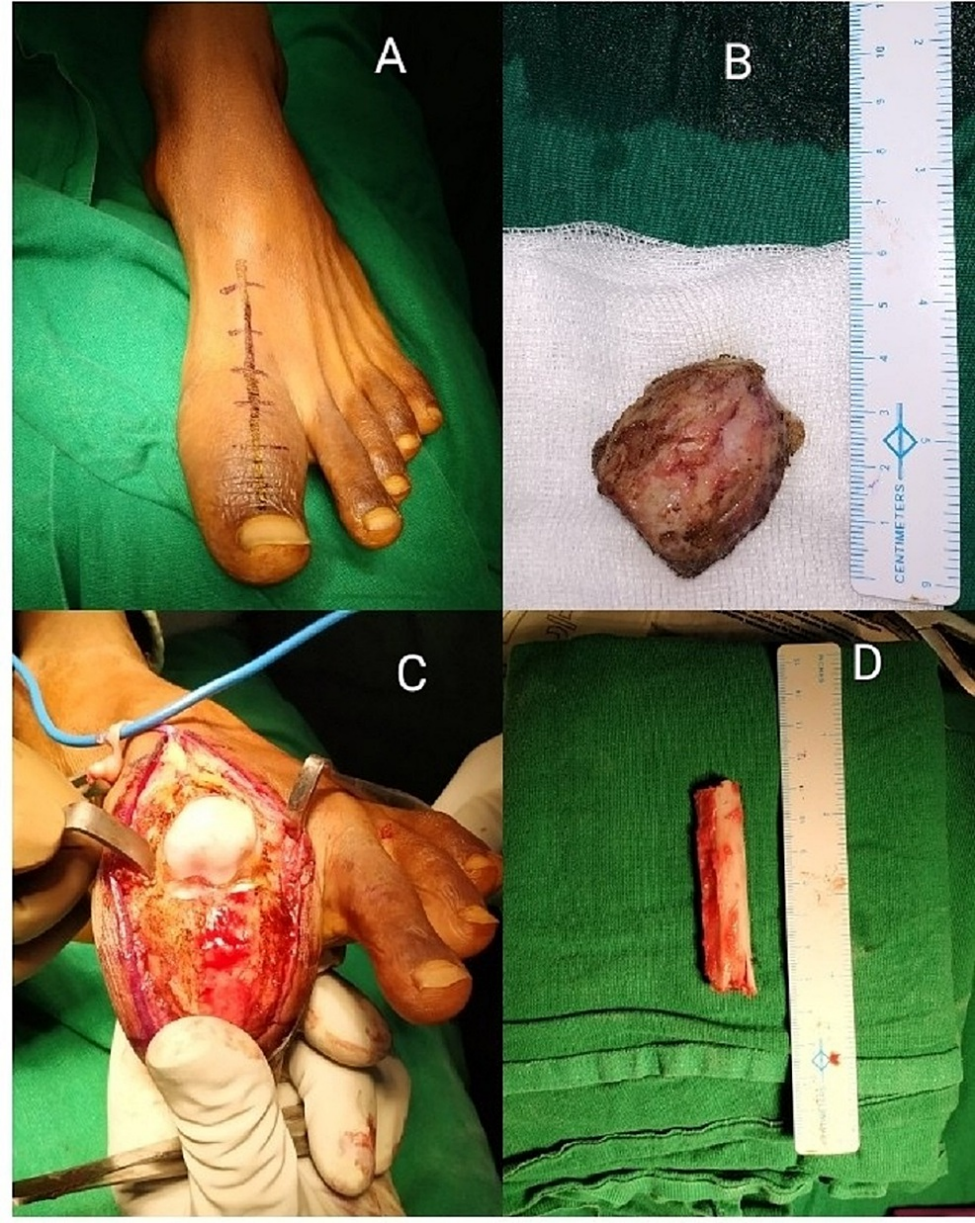
(A) Dorsal incision marked along the tendon of the extensor halluces longus. (B) Mass removed. (C) Intraoperative mass. (D) Fibular strut graft taken from the ipsilateral limb.

The dorsal incision was marked on the great toe along with the tendon of extensor halluces longus (EHL), extending 2 cm proximally to the MTP joint and distally to the base of the nail bed (Figure 3A). Complete exposure of the tumor was performed by excision of the proximal phalanx after incising the dorsal aspect of the joint capsule (Figure 3C). The swelling was expansile and multiloculated involving the whole proximal phalanx and inseparable from the skin at some areas. Hence, the decision was made to remove the whole proximal phalanx with the tumor (Figure 3B). The gap was filled up with a fibular strut graft (Figure 3D).

**Figure 4:**
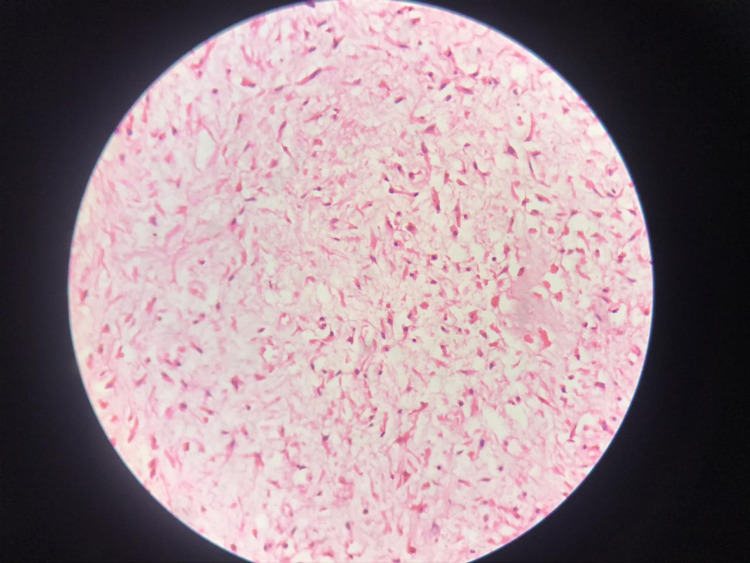
Histopathological examination of the intraoperative sample suggestive of enchondroma.

**Figure 5:**
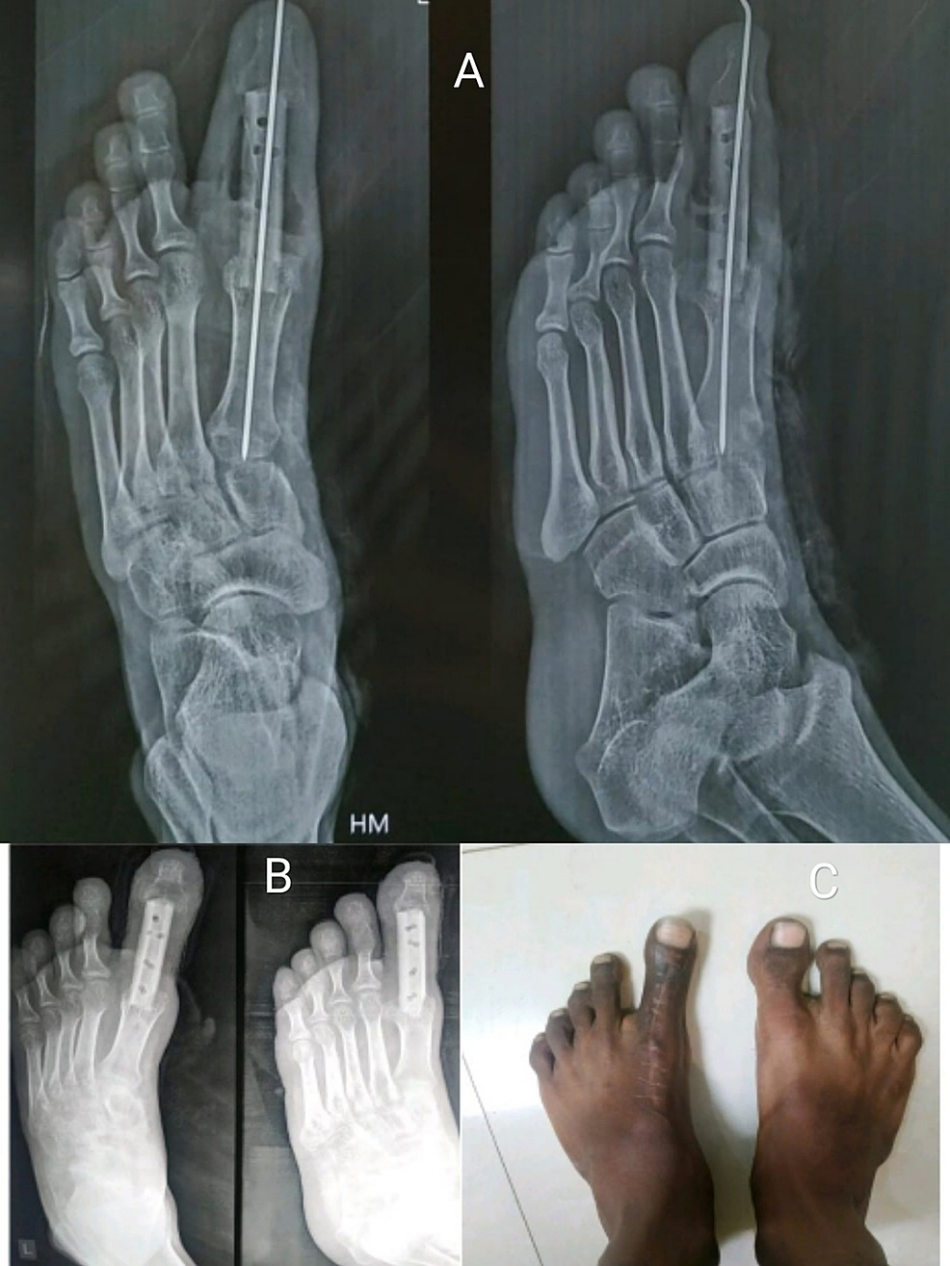
(A) Immediate postoperative X-ray. (B) Two-year postoperative follow-up X-ray. (C) Two-year follow-up clinical picture.

Histopathological examination (Figure 4) of the intraoperative sample was suggestive of enchondroma tumor comprising locules of the hyaline cartilage separated from the bone marrow. Postoperative X-ray was performed (Figure 5A), and the patient was placed on a below-knee POP (plaster of Paris) slab and non-weight-bearing for 12 weeks. Regular follow-up was performed, and an X-ray was taken at regular intervals (Figures 5B, 5C). At the two-year follow-up, the patient was fine without signs of recurrence.

## Discussion

An enchondroma is a benign tumor that develops on the bone cartilages. An enchondroma is most frequently asymptomatic, and most of the time goes unnoticed. Enchondromas are usually discovered by accident when radiographs are taken for another condition or an accident. Enchondromas can develop at any age; however, they most frequently occur in adults between 10 and 40 years of age.

The main aim of this study was to lay down management guidelines for great toe enchondroma. The significant prevalence of enchondroma in osseous structures is supported by our findings. Enchondroma is usually painful, a characteristic that has previously been reported in 40% of lesions and linked to a fracture that may be radiologically occult.

Following the clinical presentation, plain radiographs are the initial investigation of choice. On radiographs, enchondromas seem to be benign tumors with intralesional calcification. Calcification is described as “stippled” or “popcorn” in appearance. In the small bones of the hand and feet, there may be substantial erosion and growth of the overlying cortex.

MRI is the radiographic investigation of choice for confirming the lesion, which usually shows the intramedullary and soft tissue extension and any associated calcifications. Histopathological examination of tissue following biopsy is also mandatory. The diagnostic findings include visualization of lobules of hyaline cartilage, calcifications, and enchondral ossification.

If the patient develops symptoms, excision of tumor mass is an option. As done in our case, the tumor mass was excised after the patient presented with persistent complaints. Internal splinting was performed with a Kirschner wire (K-wire), and the bone defect was augmented with bone graft. The patient's lower limb was immobilized in a below-knee slab for six weeks following surgery, after which the K-wire was withdrawn and partial weight-bearing walking began. Thus, solitary enchondromas should be excised or extended curettage be performed if they are symptomatic.

Bone grafting is a widespread procedure in musculoskeletal tumor surgery, with approximately 2.2 million bone grafts performed each year to treat bone defects and around 10% of all skeletal reconstructive surgery requiring it [12]. Large defects caused by curettage of a bone tumor may necessitate the use of bone transplants (autogeneic, allogeneic, or synthetic). An autologous bone graft from the iliac crest, either cortical or cancellous, is the most recent option, but it comes with an 8%-39% complication rate (infection, hematoma, urethral injury, pelvic instability, cosmetic disadvantages, chronic pain) [13] as well as a risk of tumor cell contamination in the donor field.

Fibular strut bone graft is used for many more cases in orthopedics, such as treatment of long bone non-union, bone loss in case complex trauma, neck of femur fracture, as well as in many cases of malignancy for filling the bone gap after excision of tumor mass. In our case, fibular strut graft was used to fill the gap made after tumor mass excision.

Timely intervention by the surgeon and regular follow-up by patients resulted in good outcome and there were no signs of recurrence noted on follow-ups.

## Conclusions

Solitary enchondromas that are aggressively increasing in size should be treated surgically. The bone gap caused after removing the tumor can be filled with bone graft or cement depending on the condition of the cortex. The proximal phalangeal bone is the most common site for foot enchondroma. With appropriate treatment, a good surgical outcome can be expected. Regular follow-up is necessary for finding any signs of recurrence. Proper immobilization (at least 8-12 weeks) with POP slab of splint is important for bone healing. Good physiotherapy (knee range of motion till the period of immobilization and ankle range of motion after removal of POP slab) further adds to results postoperatively.
